# Author Correction: Fast and slow intraplate ruptures during the 19 October 2020 magnitude 7.6 Shumagin earthquake

**DOI:** 10.1038/s41467-023-44294-9

**Published:** 2023-12-14

**Authors:** Yefei Bai, Chengli Liu, Thorne Lay, Kwok Fai Cheung, Yoshiki Yamazaki

**Affiliations:** 1 Ocean College, Zhejiang University, Zhoushan, Zhejiang China; 2 Hainan Institute, Zhejiang University, Sanya, Hainan China; 3 School of Geophysics and Geomatics, China University of Geosciences, Wuhan, Hubei China; 4 Department of Earth and Planetary Sciences, University of California Santa Cruz, Santa Cruz, CA USA; 5 Department of Ocean and Resources Engineering, University of Hawaii at Manoa, Honolulu, HI USA

**Keywords:** Seismology, Natural hazards

Correction to: *Nature Communications* 10.1038/s41467-023-37731-2, published online 10 April 2023

The original version of the Supplementary Information associated with this article contained an error in the plotting of Supplementary Figs. 8d and 10d. The HTML has been updated to include a corrected version of the Supplementary Information.

### Supplementary information

Updated Supplementary Information

