# Supplementary material for: Fast and slow intraplate ruptures during the 19 October 2020 magnitude 7.6 Shumagin earthquake
Source: Nat Commun. 2023 Apr 10;14:2015. doi: 10.1038/s41467-023-37731-2 (PMC10085989; doi:10.1038/s41467-023-37731-2)
Supplement: Supplementary file 1 — Supplementary information [file 41467_2023_37731_MOESM1_ESM.pdf]

Supplementary Materials for

Fast and slow intraplate ruptures during the 19 October 2020 magnitude 7.6 Shumagin earthquake

Yefei Bai[1,2] *, Chengli Liu[3] *, Thorne Lay[4], Kwok Fai Cheung[5], Yoshiki Yamazaki[5]

* Corresponding author. Email: yfbai@zju.edu.cn, liuchengli@cug.edu.cn

This PDF file includes:

Supplementary Figs. 1 to 20
Caption for Supplementary Movie 1

Other Supplementary Materials for this manuscript include the following:

Supplementary Movie 1

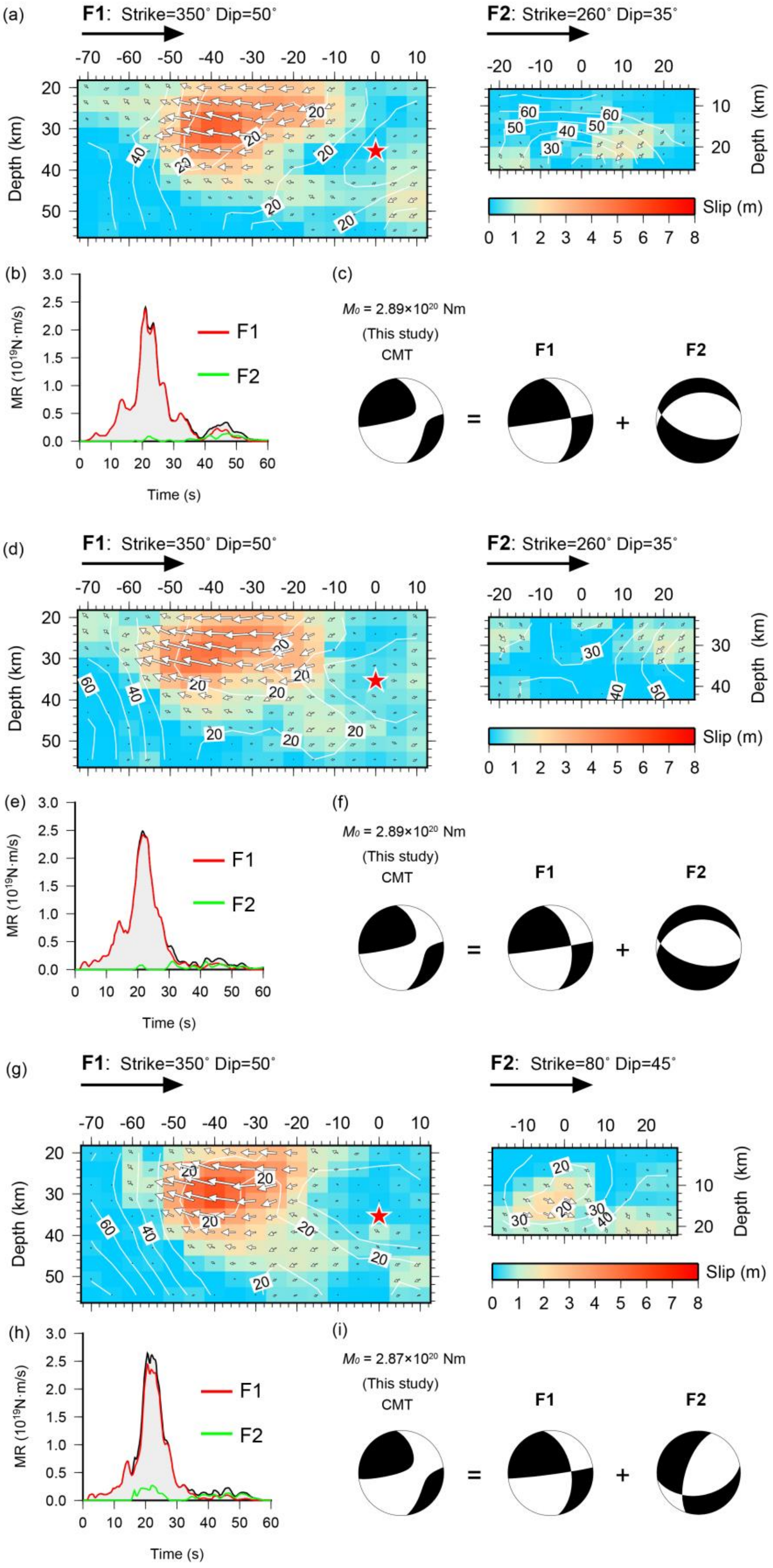
(a)
F1: Strike=350° Dip=50°
F2: Strike=260° Dip=35°
Depth (km)
Slip (m)
(b)
MR (10^19 N·m/s)
Time (s)
F1
F2
(c)
M0 = 2.89×10^20 Nm
(This study)
CMT
F1
F2
=
+
(d)
F1: Strike=350° Dip=50°
F2: Strike=260° Dip=35°
Depth (km)
Slip (m)
(e)
MR (10^19 N·m/s)
Time (s)
F1
F2
(f)
M0 = 2.89×10^20 Nm
(This study)
CMT
F1
F2
=
+
(g)
F1: Strike=350° Dip=50°
F2: Strike=80° Dip=45°
Depth (km)
Slip (m)
(h)
MR (10^19 N·m/s)
Time (s)
F1
F2
(i)
M0 = 2.87×10^20 Nm
(This study)
CMT
F1
F2
=
+

Supplementary Fig. 1. Two-fault fast-slip models with shallow northward dipping fault F2 (a)-(c), intraslab northward dipping fault F2 (d)-(f), and shallow southward dipping fault F2 (g)-(i) for the 19 October 2020 M_W 7.6 Shumagin Earthquake. (a) Slip models for faults F1 and F2 for (a) the shallow northward-dipping F2, (d) the intraslab northward-dipping F2, and (g) shallow southward-dipping F2. Moment-rate function (gray-shaded region) for the model with (b) shallow northward-dipping F2, and (e) intraslab northward-dipping F2, and (h) shallow southward-dipping F2; red and green curves indicate the contribution from the two fault segments. The total moment tensor, and separate moment tensors for F1 and F2 for (c) the shallow northward-dipping F2, (f) the intraslab northward-dipping F2, and (i) the shallow southward-dipping F2. The slip models are from joint inversion of teleseismic *P* and *SH* ground velocities, regional three-component broadband and strong-motion ground velocities, regional geodetic static offsets and high-rate GNSS displacement time series. The red stars locate the hypocenter on fault plane F1. White contours indicate the rupture initiation time in seconds from the origin. White arrows show the variable direction and magnitude of the slip. The color pattern shows the slip scale. The model in (a) is shown in map view in Fig. 3a, that in (d) is shown in Supplementary Fig. 2c, and that in (g) is shown in Supplementary Fig. 2f.

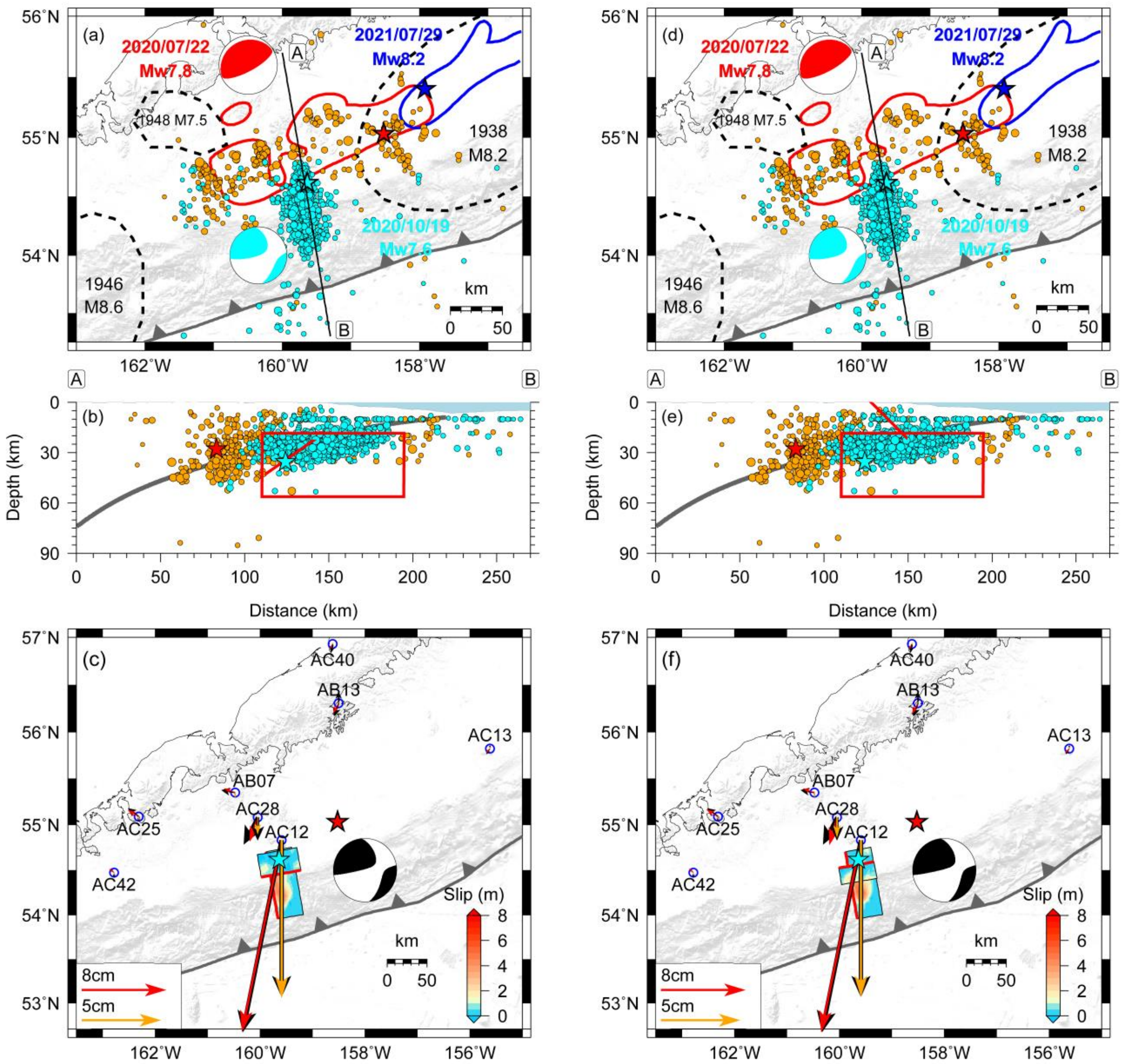

Supplementary Fig. 2. (a) Map similar to Fig 1a, but indicating the geometry of the two-fault fast-slip model with intraslab northward-dipping F2 in Supplementary Fig. 1d. (b) cross-section similar to Fig. 1b, but indicating the intraslab northward-dipping F2. (c) Map similar to Fig. 3a showing the co-seismic offsets at regional GNSS stations and position of the slip model for the intraslab northward-dipping F2 case in Supplementary Fig. 1d. (d) Map similar to Fig 1a, but indicating the geometry of the two-fault fast-slip model with shallow southward-dipping F2 in Supplementary Fig. 1g. (e) cross-section similar to Fig. 1b, but indicating the shallow southward-dipping F2. (f) Map similar to Fig. 3a showing the co-seismic offsets at regional GNSS stations and position of the slip model for the shallow southward-dipping F2 case in Supplementary Fig. 1g.

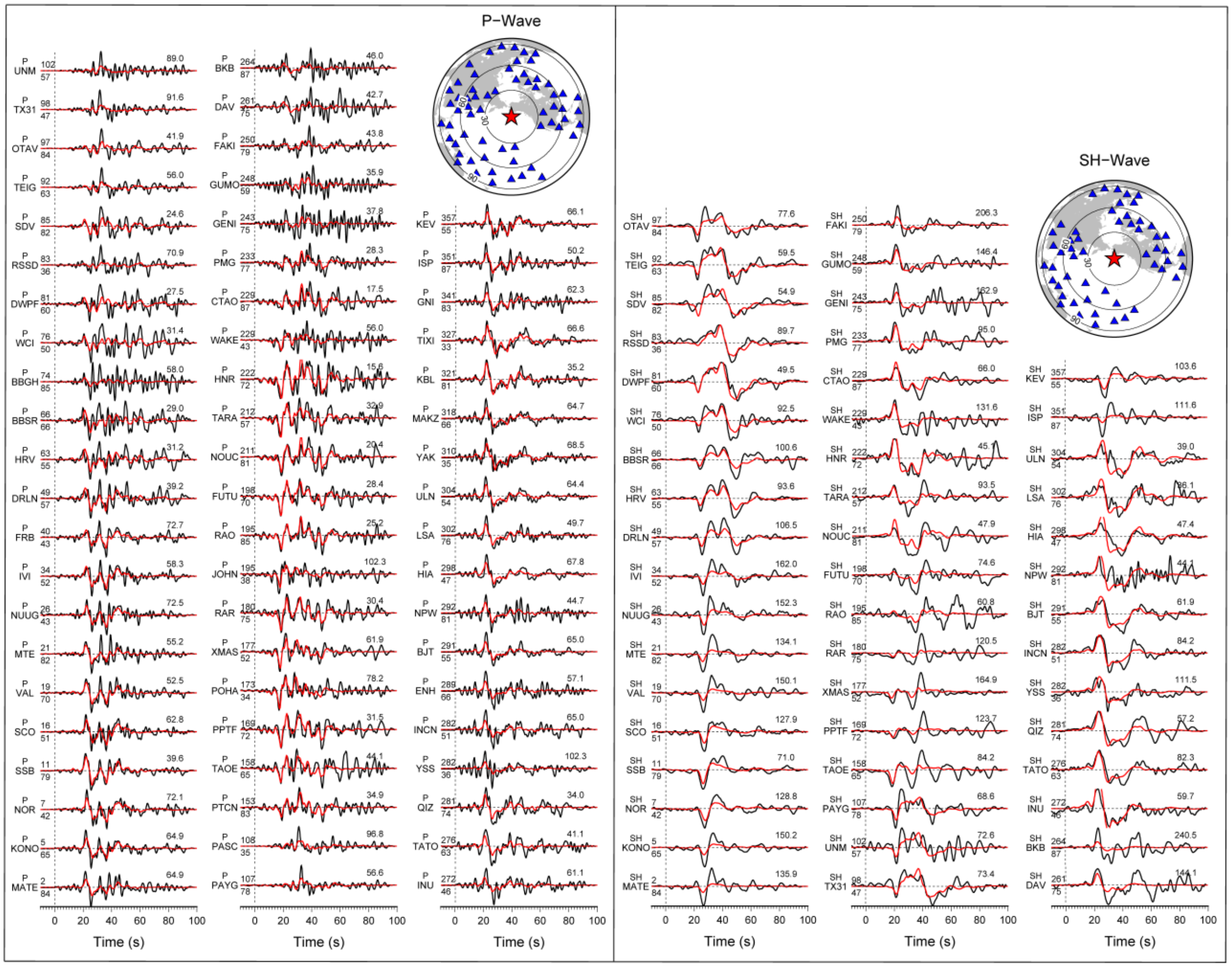

Supplementary Fig. 3. Teleseismic waveform fits for the preferred two-fault fast-slip model with shallow north-dipping F2 for the 19 October 2020 Shumagin M_W 7.6 earthquake. Comparison of observed (black) and synthetic (red) teleseismic *P* (let panel) and *SH* (right panel) wave ground velocities for the fast-slip model with two fault segments in Fig. 3a and Supplementary Fig. 1a. Data and synthetic seismograms are manually aligned on the first arrivals. Station names and phase-type are indicated on the left of each comparison. The number above the right portion of each comparison is the peak amplitude of the observed ground velocity in μm/s. The azimuth (above) and distance (below) in degrees are shown at the beginning of each record. The station distribution (blue triangles) nearby the epicenter (red star) is located at the upper right corner of each panel.

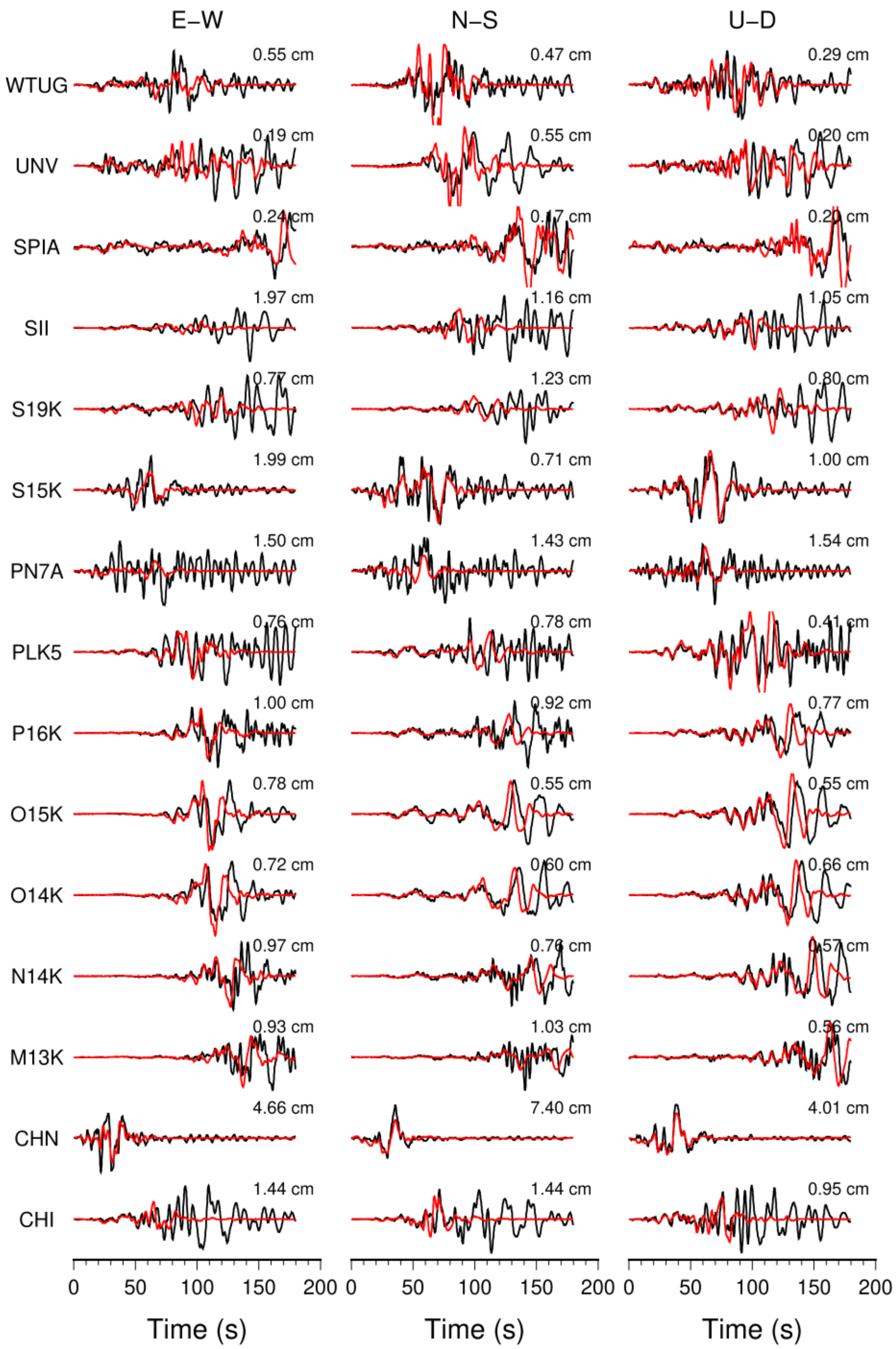

Supplementary Fig. 4. Regional broadband and strong-motion waveform fits for the preferred two-fault fast-slip model with shallow north-dipping F2 for the 19 October 2020 Shumagin M_W 7.6 earthquake. Comparison of observed three-component broadband and strong-motion ground-velocity records (black lines) and synthetic seismograms (red lines) for the slip model with two fault segments in Fig. 3a and Supplementary Fig. 1a. Data and synthetics are manually aligned on the first *P* wave arrival. Station names are indicated on the left; the number at the top right of each trace comparison is the maximum displacement of the observed signals in cm/s.

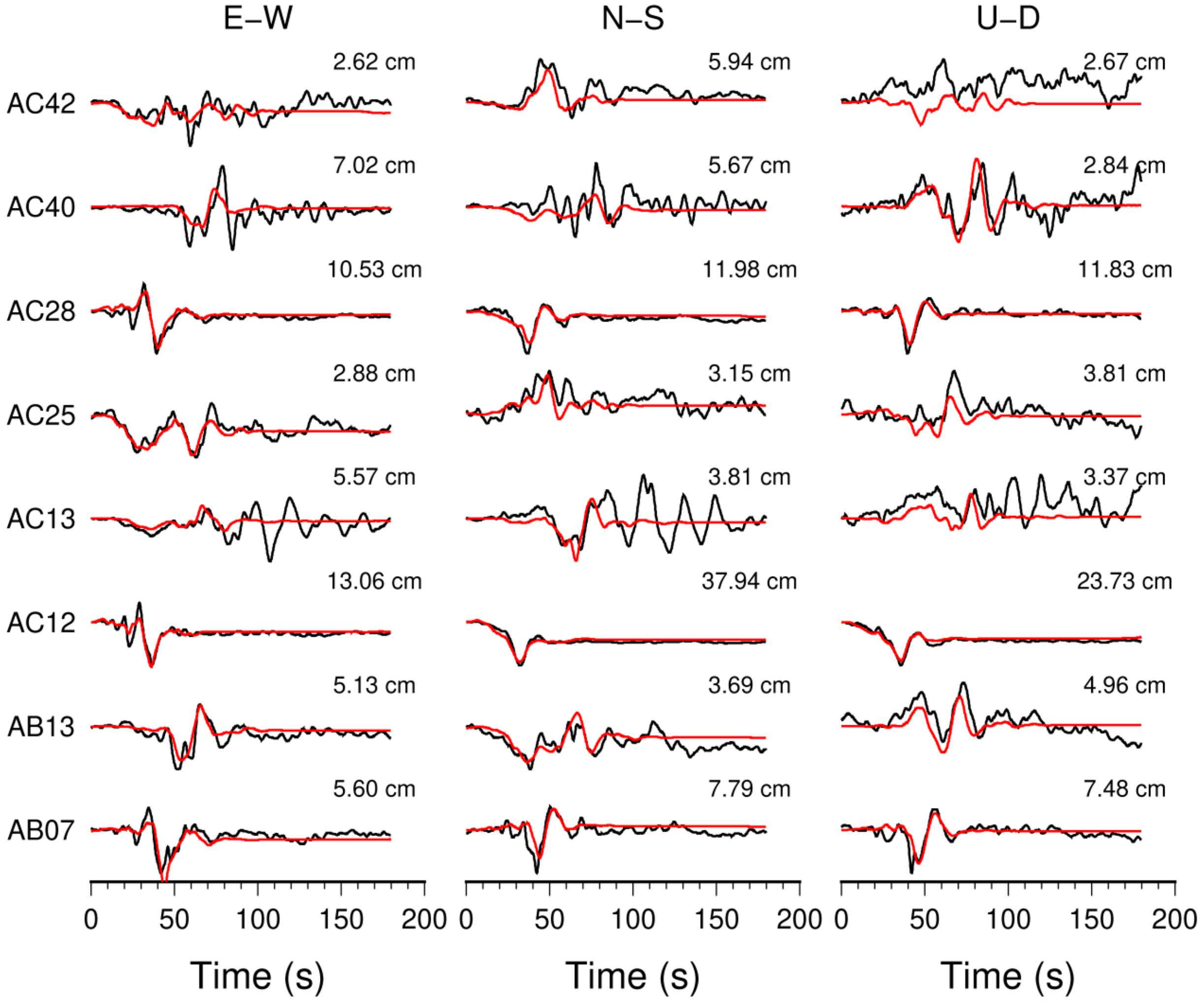

Supplementary Fig. 5. Hr-GNSS waveform fits for the preferred two-fault fast-slip model with shallow north-dipping F2 for the 19 October 2020 Shumagin M_W 7.6 earthquake. Comparison of observed three-component hr-GNSS ground displacement records (black lines) and synthetic seismograms (red lines) for the slip model with two fault segments in Fig. 3a and Supplementary Fig. 1a. Data and synthetics are manually aligned on the first *P* wave arrival. Station names are indicated on the left; the number at the top right of each trace comparison is the maximum ground displacement of the observed signals in cm.

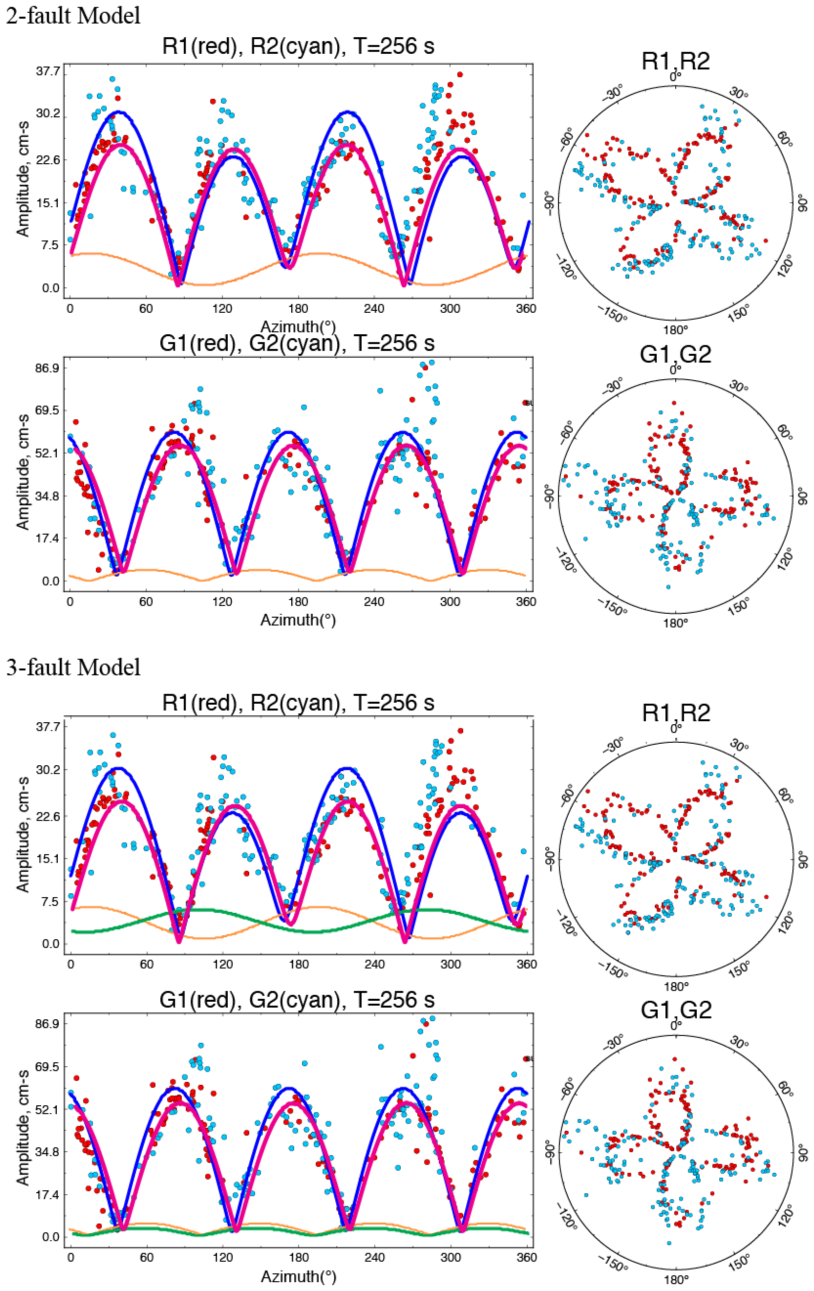

Supplementary Fig. 6. Long-period surface wave spectra for the 19 October 2020 Shumagin M_W 7.6 earthquake. Observed (dots) and predicted (curves) 256-s Rayleigh and Love wave spectral amplitudes for short-arc (R1, G1, red dots) and long-arc (R2, G2, cyan dots) paths, corrected to the source. Data are shown both on the left and in the rose diagrams on the right. The two-fault fast-slip model with shallow north-dipping F2 (Fig. 3a and Supplementary Fig. 1a) predictions are shown in the top two rows for the strike-slip fault (blue), and normal-fault (red) faulting separately, and their phase-weighted sum (magenta). The same curves are shown for the three-fault model (Fig. 7) in the lower two rows, with the green curve showing the pattern expected for a 300 s long dislocation on the upper plate thrust fault. This is not added to the total motion, due to large phase shift, but the amplitudes for R1 and G1 are negligible at this period.

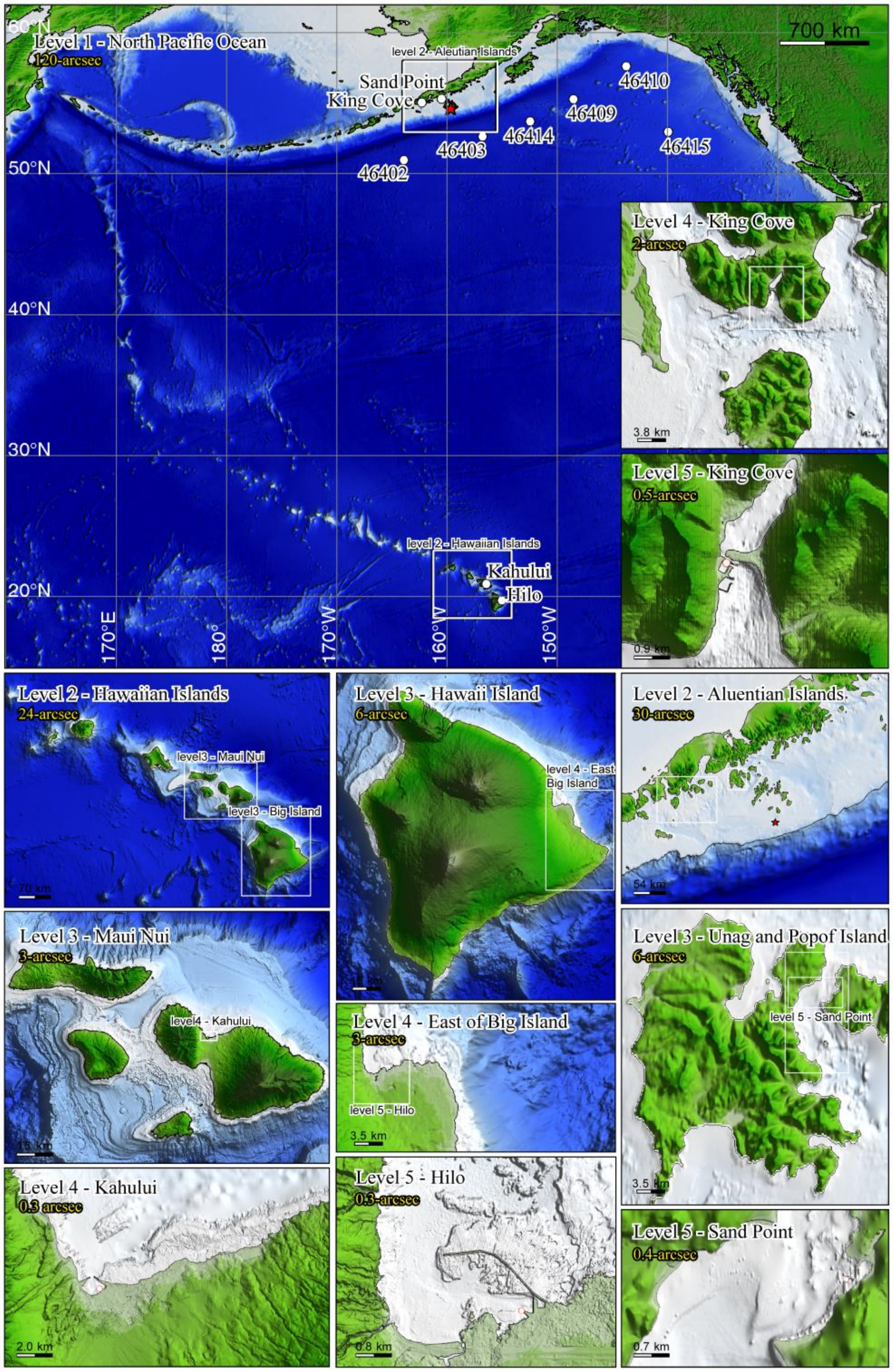

Supplementary Fig. 7. Multi-level computational grids for tsunami modeling. The Kahului tide gauge is modeled with four levels of computational grids and Hilo and Sand Point are modeled with five levels due to the more complex coastal bathymetry. The grid resolution is shown in each panel. Red star indicates the location of the 19 October 2020 epicenter and white circles denote tide gauge and DART locations.

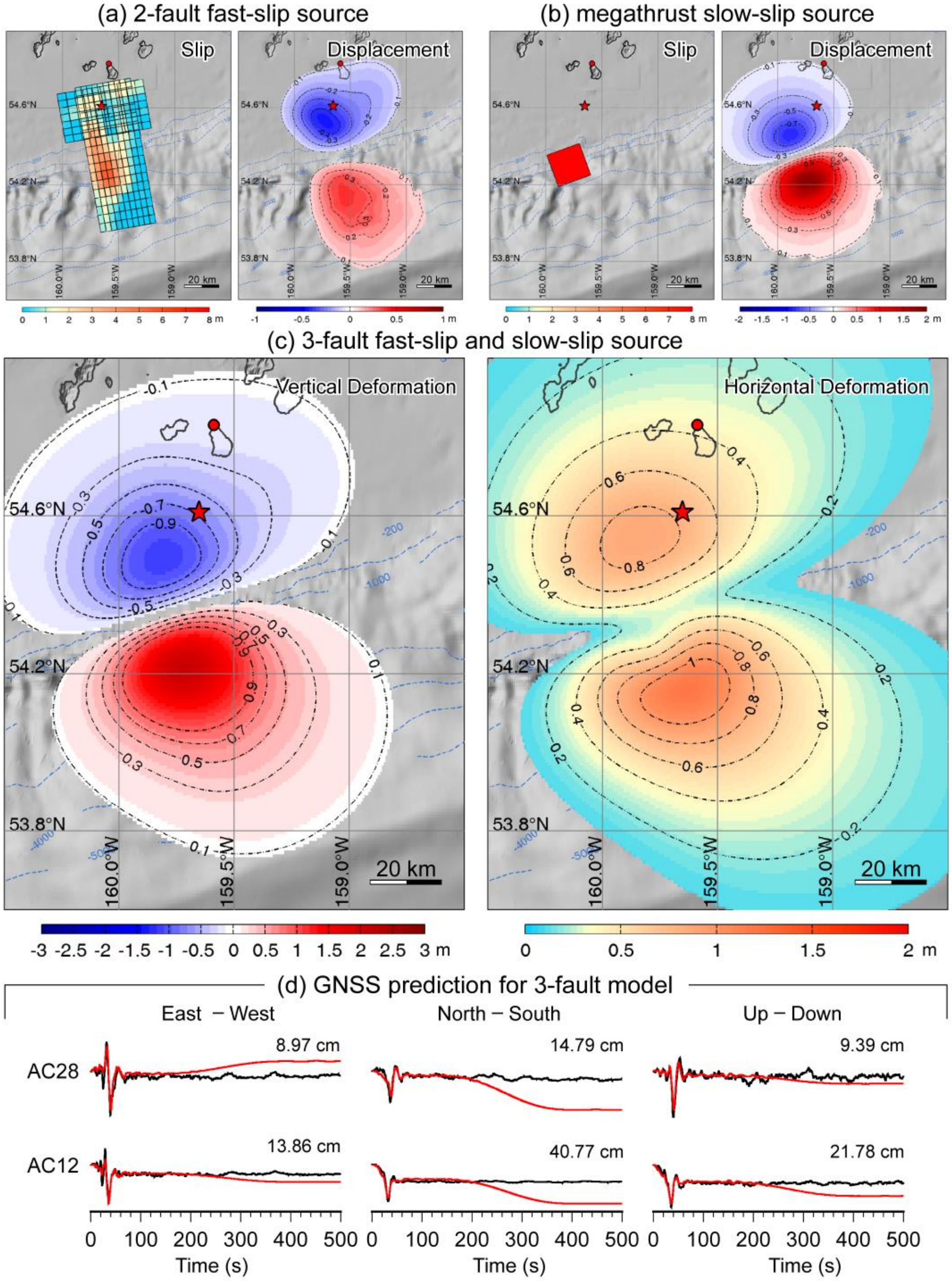

Supplementary Fig. 8. Slip and seafloor deformation for (a) the preferred two-fault fast-slip model with shallow north-dipping F2 (Figure 3a)**;** and (b) an additional slow-slip megathrust thrust faulting source. (c) The superimposed vertical and horizontal seafloor deformation from the combined three-fault fast-slip and slow-slip model. (d) Observed (black) and computed (red) hr-GNSS ground motions for stations AC28 and AC12 extending over a 600 s time scale. Tsunami predictions for this model are shown in Supplementary Fig. 9. Red stars indicate the epicenter, and red circles denote GNSS station AC12.

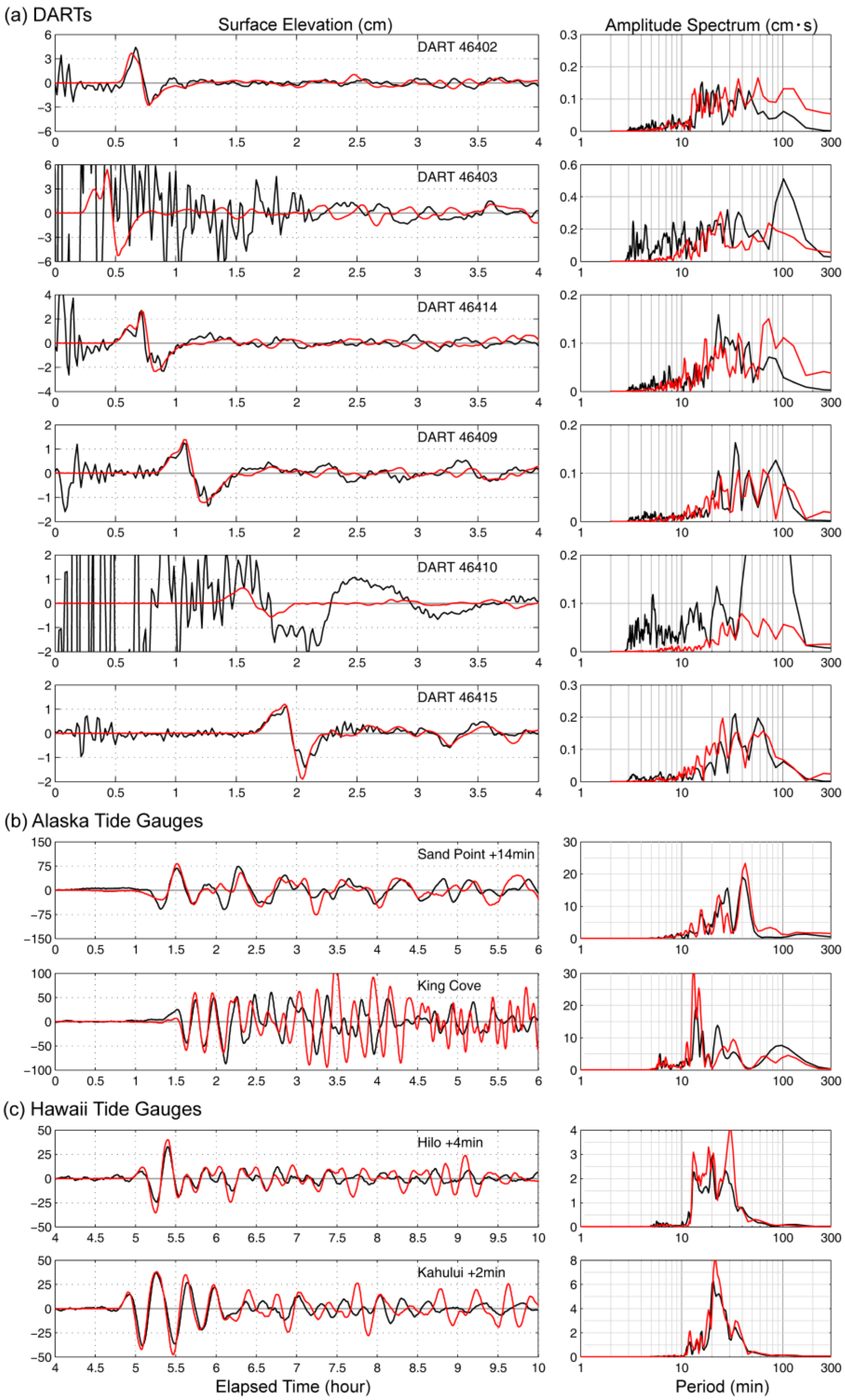

Supplementary Fig. 9. Tsunami predictions for the three-fault model with fast-slip and slow-slip megathrust thrust faulting in Supplementary Fig. 8. Observed (black lines) and predicted (red lines) tsunami surface elevation time series (left column) and spectra (right column) for the three-fault model. (a) DART stations. (b) Alaska tide gauges. (c) Hawaii tide gauges. The computed time series at the tide gauges have been shifted by the indicated times to align with the recorded arrival.

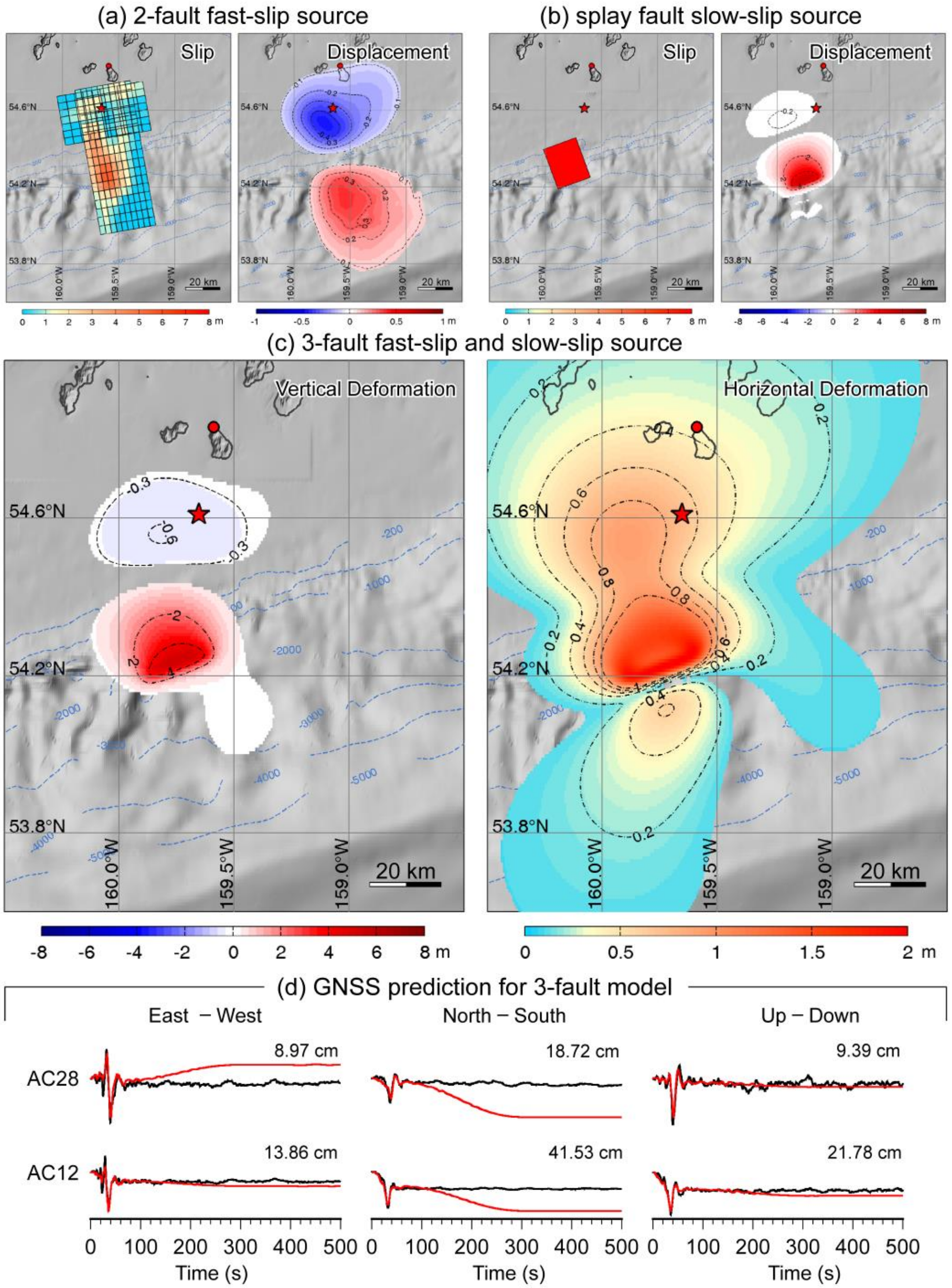

Supplementary Fig. 10. Slip and seafloor deformation for (a) the preferred two-fault fast-slip model with shallow north-dipping F2 (Figure 3a), and (b) an additional slow-slip splay faulting thrust source. (c) The superimposed vertical and horizontal seafloor deformation from the combined three-fault fast-slip and slow-slip splay fault model is shown in the center panels. Observed (black) and computed (red) hr-GNSS ground motions for stations AC28 and AC12 extending over a 600 s time scale are shown in the lower panels. Tsunami predictions for this model are shown in Supplementary Fig. 11. Red stars indicate the epicenter, and red circles denote GNSS station AC12.

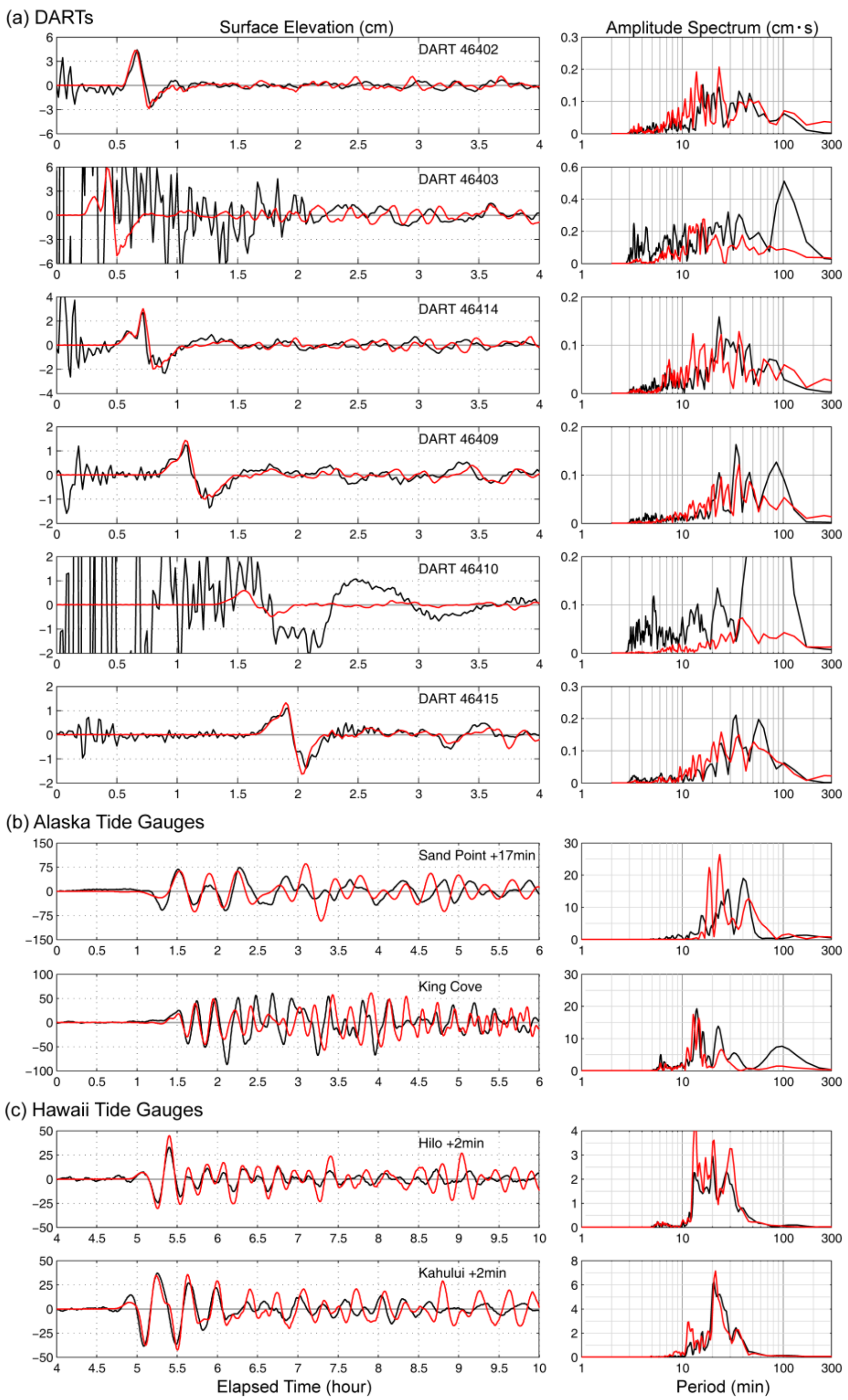

Supplementary Fig. 11. Tsunami predictions for the three-fault model with slow splay faulting in Supplementary Fig. S10. Observed (black lines) and predicted (red lines) tsunami surface elevation time series (left column) and spectra (right column) for the three-fault model. (a) DART stations. (b) Alaska tide gauges. (c) Hawaii tide gauges. The computed time series at the tide gauges have been shifted by the indicated times to align with the recorded arrival.

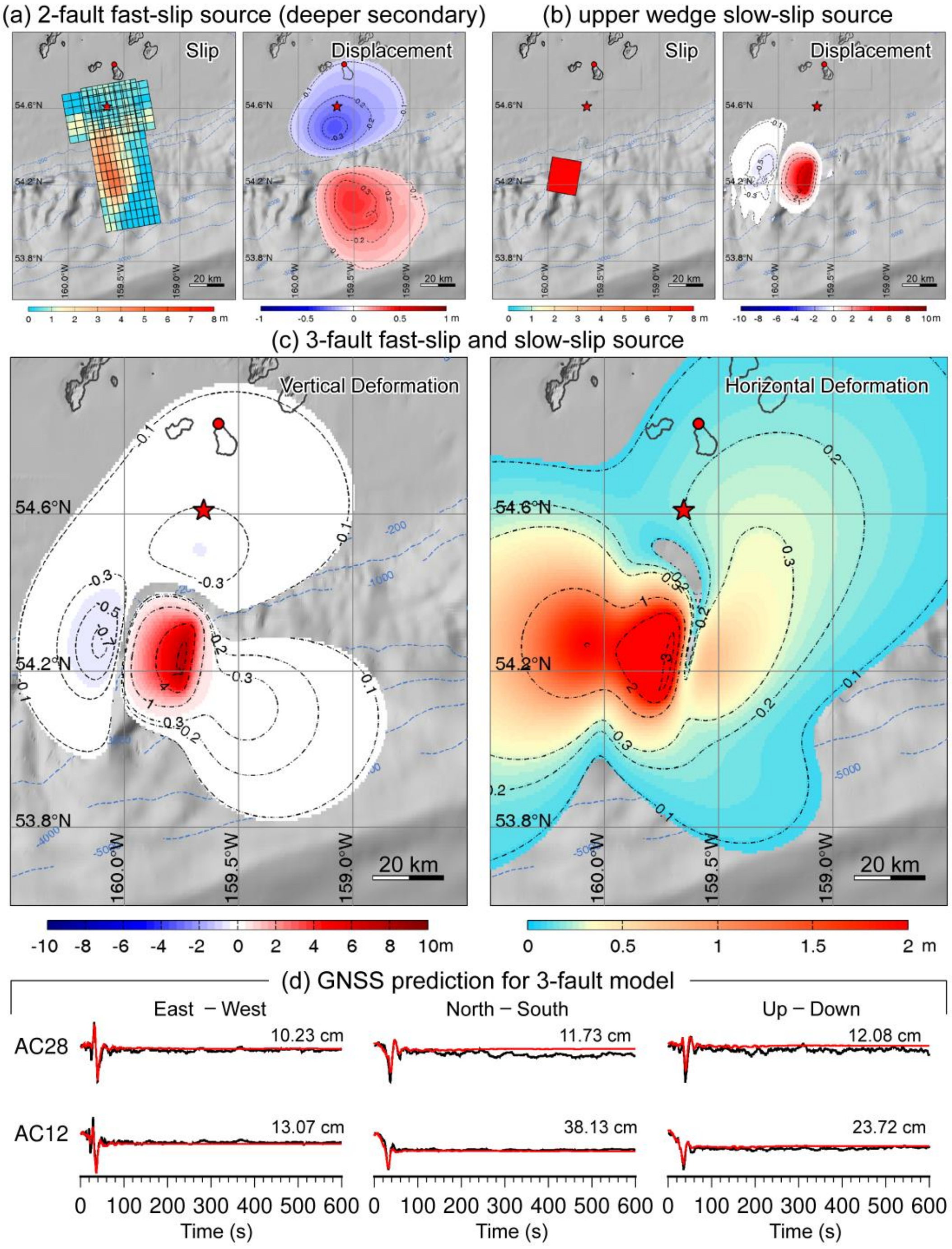

Supplementary Fig. 12. Slip and seafloor deformation for (a) the two-fault fast-slip model with intraslab north-dipping F2 (Supplementary Fig. 2c), and (b) an additional upper plate slow-slip faulting. (c) The superimposed vertical and horizontal seafloor deformation from the combined three-fault fast-slip and slow-slip fault model. (d) Observed (black) and computed (red) hr-GNSS ground motions for stations AC28 and AC12 extending over a 600 s time scale are shown in the lower panels. Tsunami predictions for this model are shown in Supplementary Fig. 13. Red stars indicate the epicenter, and red circles denote GNSS station AC12.

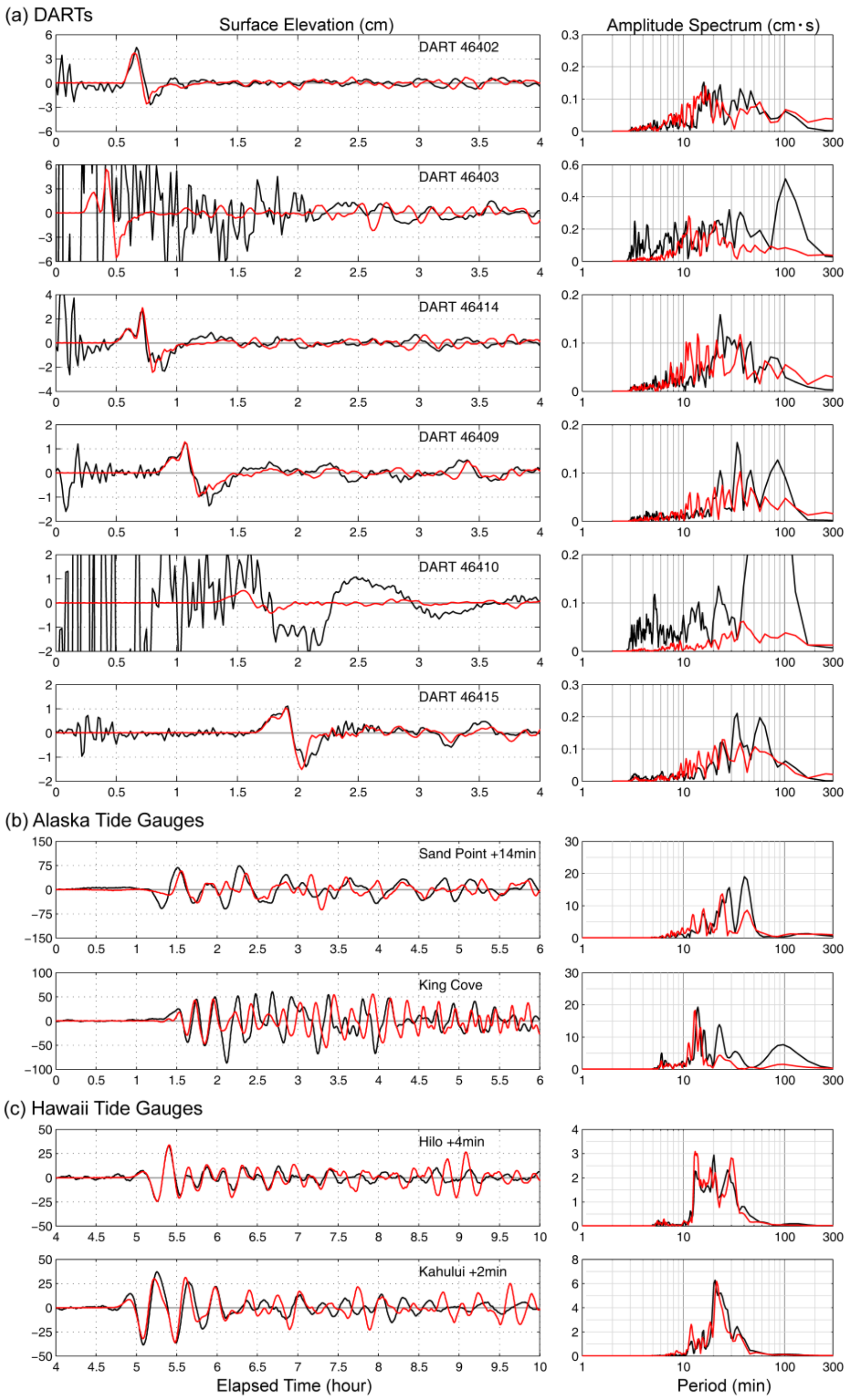

Supplementary Fig. 13. Tsunami predictions for the three-fault model with slow faulting in Supplementary Fig. S12. Observed (black lines) and predicted (red lines) tsunami surface elevation time series (left column) and spectra (right column) for the three-fault model. (a) DART stations. (b) Alaska tide gauges. (c) Hawaii tide gauges. The computed time series at the tide gauges have been shifted by the indicated times to align with the recorded arrival.

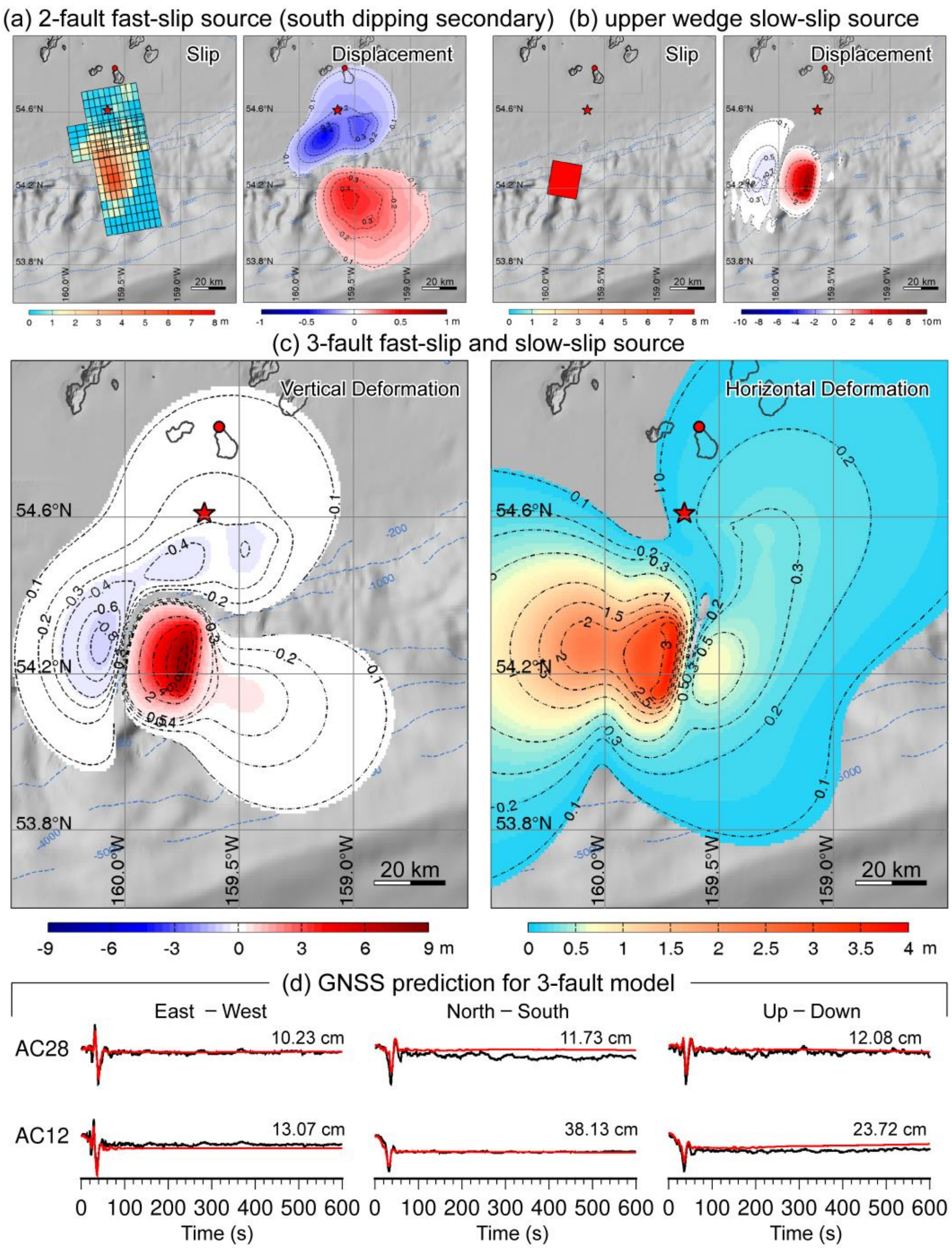

Supplementary Fig. 14. Slip and seafloor deformation for (a) the two-fault fast-slip model with shallow south-dipping F2 and (b) an additional upper plate slow-slip faulting (upper right panels). (c) The superimposed vertical and horizontal seafloor deformation from the combined three-fault fast-slip and slow-slip fault model is shown in the center panels. (d) Observed (black) and computed (red) hr-GNSS ground motions for stations AC28 and AC12 extending over a 600 s time scale are shown in the lower panels. Tsunami predictions for this model are shown in Supplementary Fig. 15. Red stars indicate the epicenter, and red circles denote GNSS station AC12.

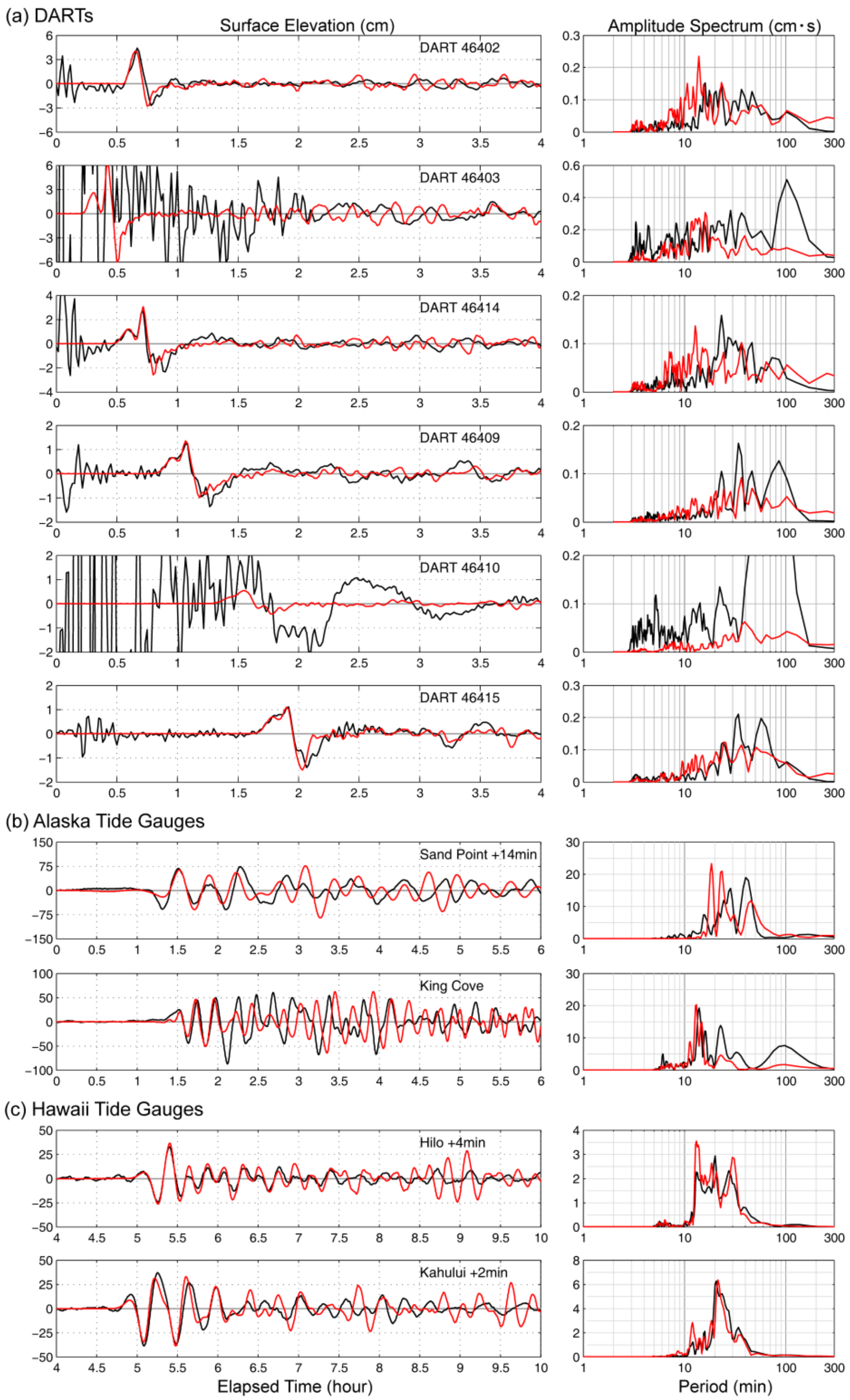

Supplementary Fig. 15. Tsunami predictions for the three-fault model in Supplementary Fig. S14. Observed (black lines) and predicted (red lines) tsunami surface elevation time series (left column) and spectra (right column) for the three-fault model. (a) DART stations. (b) Alaska tide gauges. (c) Hawaii tide gauges. The computed time series at the tide gauges have been shifted by the indicated times to align with the recorded arrival.

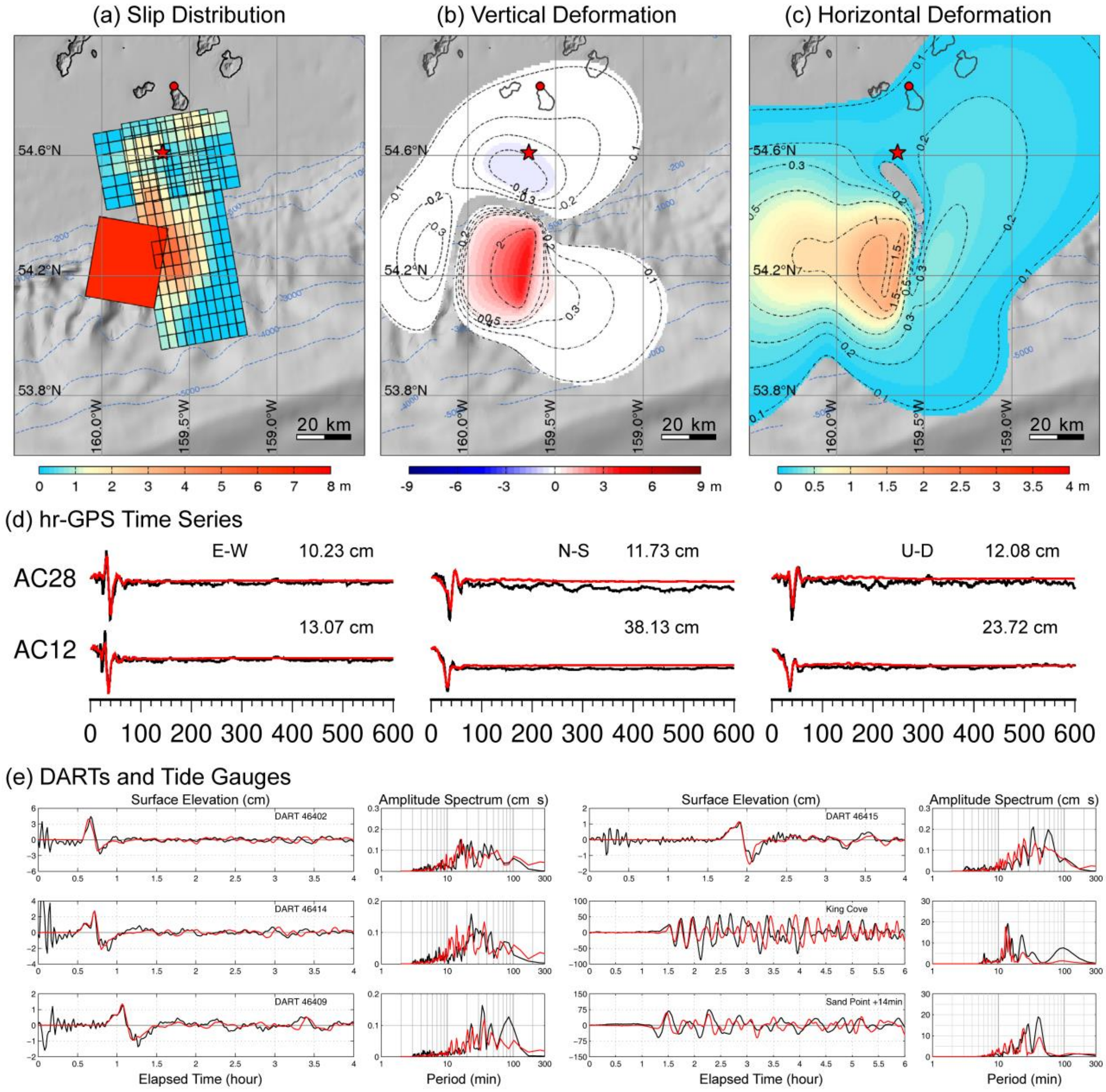

Supplementary Fig. 16. Seafloor deformation, GNSS ground motion, and associated tsunami predictions for the combined fast- and 30 km by 30 km medium slip (7 m) slow-slip model. (a) Slip distribution, (b) vertical deformation, and (c) horizontal deformation of the combined 3-fault model. (d) Observed (black) and computed (red) GNSS ground motions for stations AC28 and AC12 extending over a 600s time scale. Red star indicates the epicenter, and the red circles denotes GNSS station AC12. (e) Observed (black) and predicted 3-fault (red) tsunami surface elevation time series (left column) and spectra (right column) for selected DART and tide gauge stations.

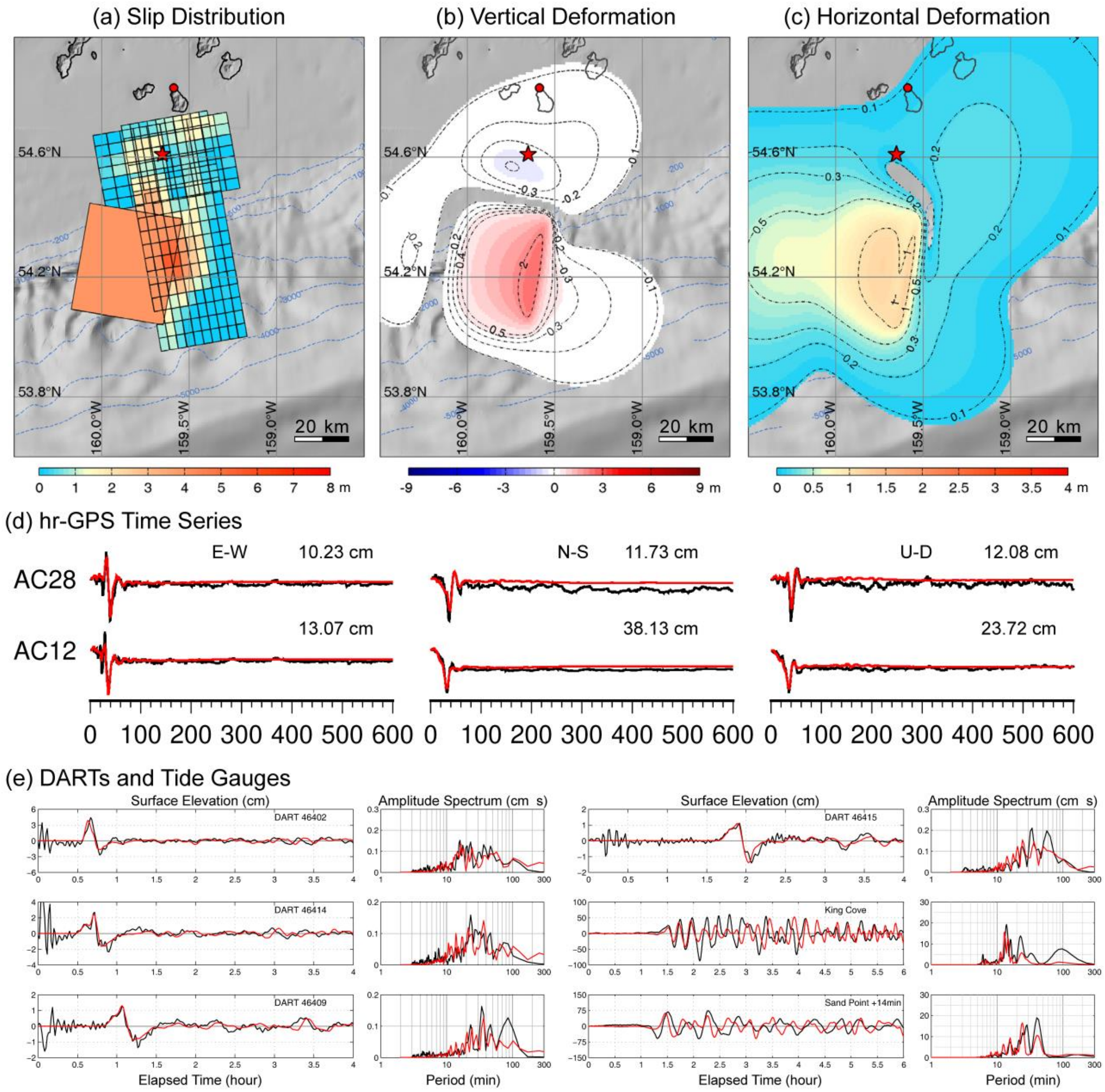

Supplementary Fig. 17. Seafloor deformation, GNSS ground motion, and associated tsunami predictions for the combined fast- and with 40 km x 40 km low slip (4 m) slow-slip model. (a) Slip distribution, (b) vertical deformation and (c) horizontal deformation of the combined 3-fault model. (d) Observed (black) and computed (red) GNSS ground motions for stations AC28 and AC12 extending over a 600s time scale. Red star indicates the epicenter, and the red circles denotes GNSS station AC12. (e) Observed (black) and predicted 3-fault (red) tsunami surface elevation time series (left column) and spectra (right column) for selected DART and tide gauge stations.

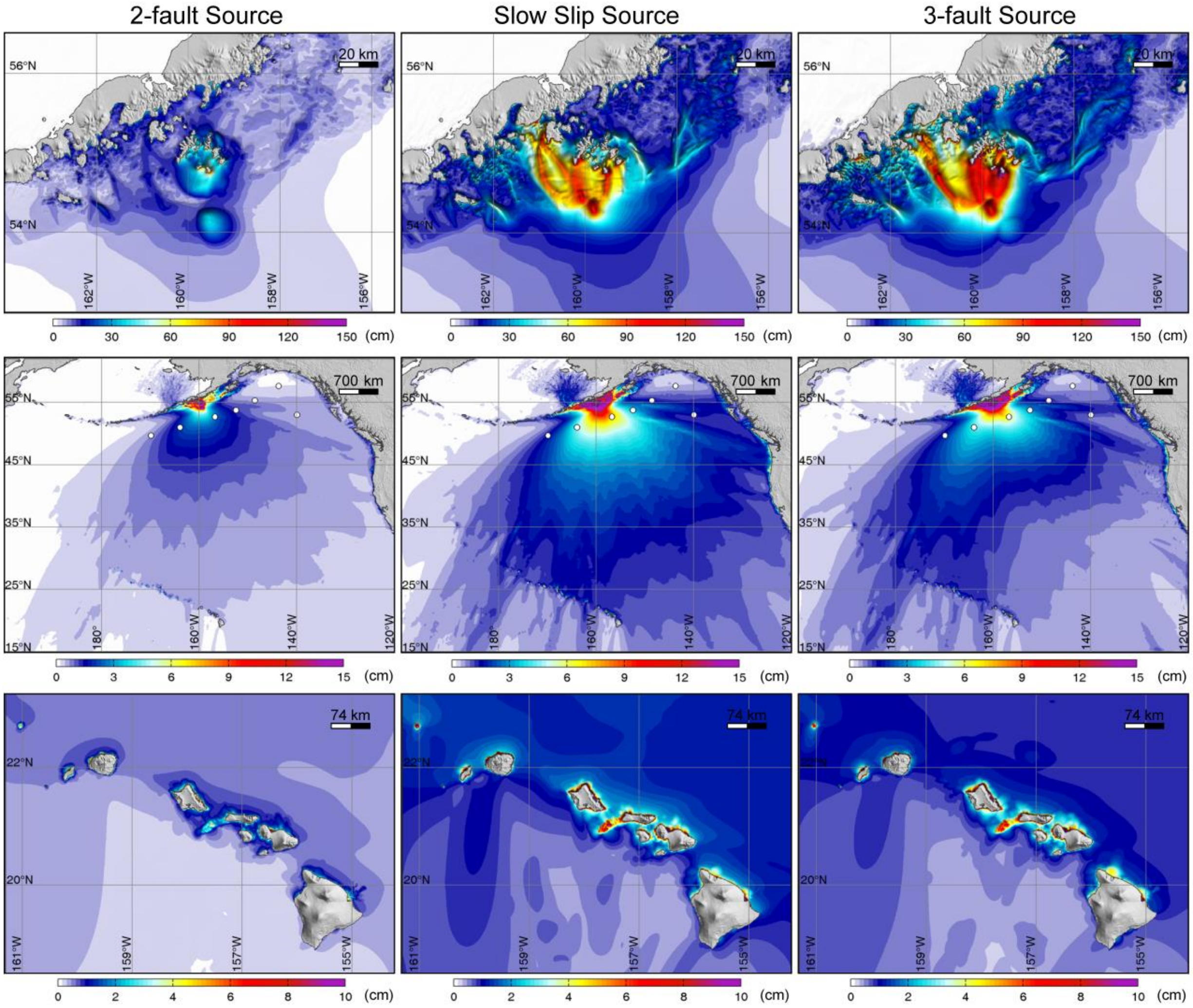

Supplementary Fig. 18. Peak tsunami wave amplitudes for near-field (top row), north-Pacific (middle row) and near-Hawaii (lower row) computations for the two-fault fast-slip model in Fig. 3a (left column), upper plate slow-slip thrust fault in Fig. 7 (center column), and combined three-fault model in Fig. 7 (right column). White circles denote locations of the DART stations.

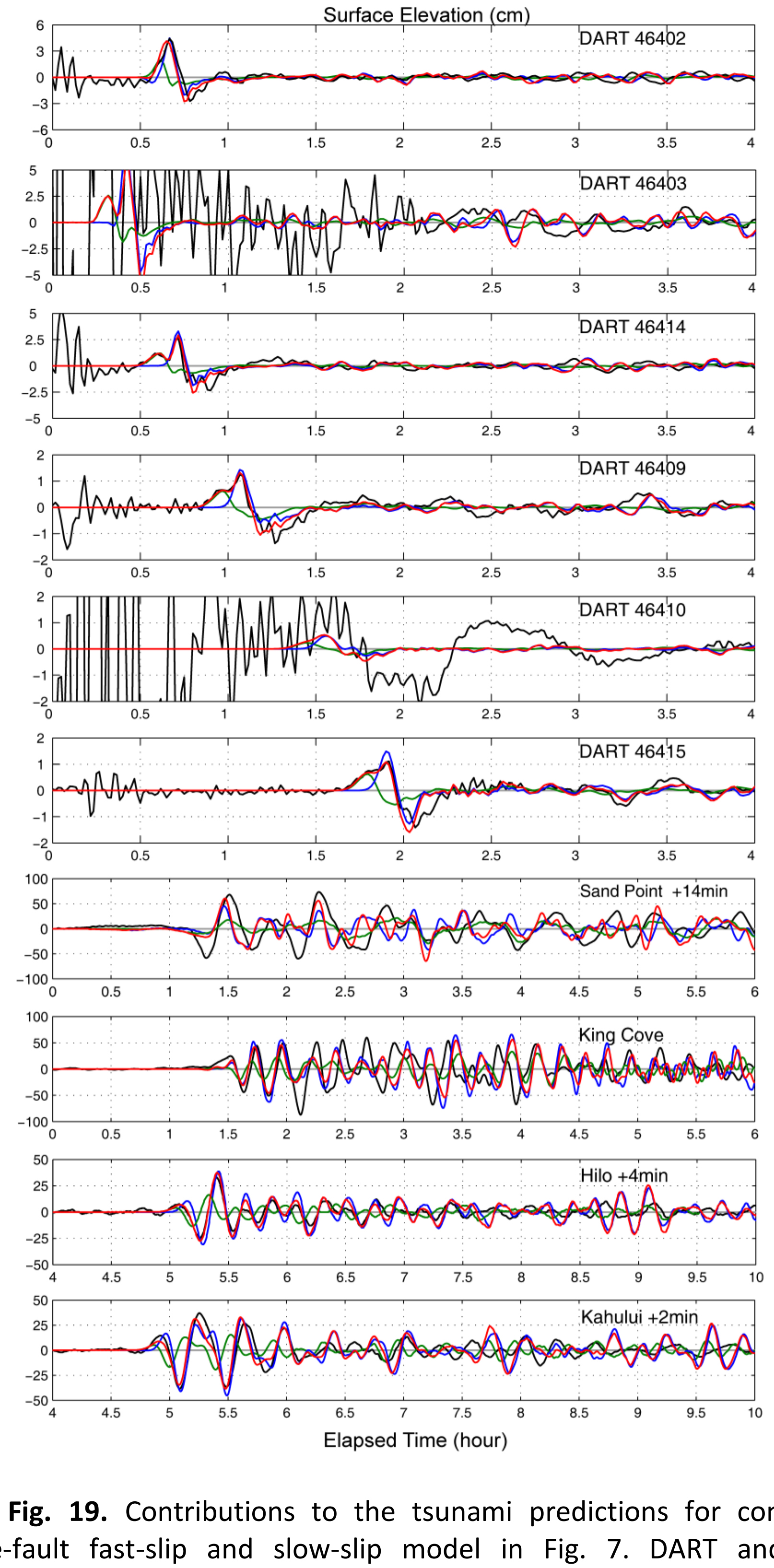

Supplementary Fig. 19. Contributions to the tsunami predictions for components of the preferred three-fault fast-slip and slow-slip model in Fig. 7. DART and tide gauge fit decompositions: two-fault fast-slip waveform (green), slow-slip thrust fault contribution (blue), total combined wave (red).

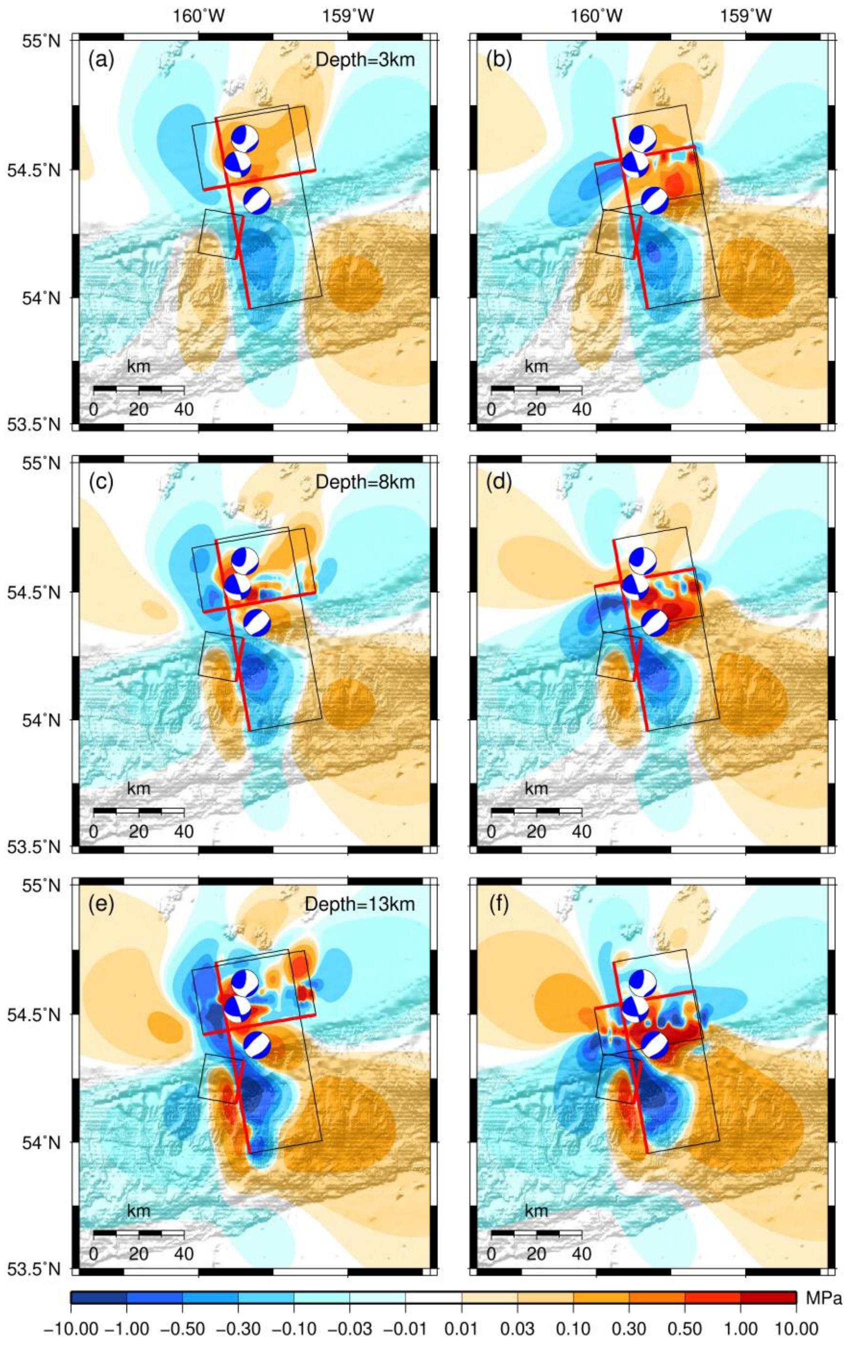

Supplementary Fig. 20. Maps showing calculated Coulomb stress change on the geometry of the slow-slip thrust fault for the two fast-slip models with northward-dipping F2 (left column) and southward dipping F2 (right column). The boxes outline the fast-slip fault geometries, with the up-dip edge of faults highlighted in red. The westward-dipping fault is the preferred location of the slow-slip event. The region around this fault has modest Coulomb stress increases of up to 0.5 MPa for both fast-slip models, consistent with triggered slow slip. GCMT focal mechanisms for 3 M_W 4.8-4.9 aftershocks are shown, indicative of complex extensional stress in the upper plate under the shelf where fast-faulting occurred.
