# Supplementary material for: Fast and slow intraplate ruptures during the 19 October 2020 magnitude 7.6 Shumagin earthquake
Source: Nat Commun. 2023 Apr 10;14:2015. doi: 10.1038/s41467-023-37731-2 (PMC10085989; doi:10.1038/s41467-023-37731-2)
Supplement: Supplementary file 3 — Description of Additional Supplementary files [file 41467_2023_37731_MOESM3_ESM.pdf]

Description of Additional Supplementary Files

File name: Supplementary Movie 1
Description: Tsunami wave field for the Shumagin region based on the preferred three-fault fast-slip and slow-slip model (Fig. 7).
